# Effect of routine seasonal malaria chemoprevention on malaria trends in children under 5 years in Dangassa, Mali

**DOI:** 10.1186/s12936-020-03202-y

**Published:** 2020-04-06

**Authors:** Drissa Konaté, Sory I. Diawara, Mahamoudou Touré, Seidina A. S. Diakité, Agnès Guindo, Karim Traoré, Ayouba Diarra, Bourama Keita, Sibe Thiam, Moussa Keita, Ibrahim Sissoko, Nafomon Sogoba, Sékou F. Traoré, Donald J. Krogtad, Seydou Doumbia, Mahamadou Diakité

**Affiliations:** 1 West African International Center for Excellence in Malaria Research (ICEMR-WA), University of Sciences, Techniques and Technologies of Bamako, Bamako, Mali; 2 University Clinical Research Center, University of Sciences, Techniques and Technologies of Bamako, Bamako, Mali; 3 Tulane University School of Public Health and Tropical Medicine, New Orleans, LA USA

**Keywords:** Children, Malaria indicators, SMC, NMCP, Mali

## Abstract

**Background:**

Seasonal malaria chemoprevention (SMC) is a new strategy to prevent malaria in children under 5 years old. It has been recommended by the World Health Organization since 2012 in malaria-endemic areas with seasonal transmission. This study aimed to assess the changes in malaria indicators through two consecutive years of SMC routine implementation in children under 5 years old in Dangassa, where malaria is endemic with a long and high transmission season.

**Methods:**

From 2012 to 2016, a cohort study was conducted in Dangassa village. The study team based in the village followed all malaria clinical cases in children under 5 years old at the community health centre. During the study, SMC was routinely implemented in collaboration with the National Malaria Control Programme. The Cox regression model was used in order to compare malaria risk during the study.

**Results:**

The Cox regression model showed a significant reduction in malaria clinical incidence, both in 2015 (HR = 0.27 (0.18–0.40), 95% CI) and in 2016 (HR = 0.23 (0.15–0.35), 95% CI) of SMC implementation compared to October 2013. Gametocyte and fever prevalence was lower between September and October during SMC implementation (2015 and 2016) compared to the same period before SMC implementation (2013–2014). A slight increase of malaria incidence was observed in December at the end of SMC implementation.

**Conclusion:**

SMC has significantly reduced both malaria incidence and gametocyte prevalence and improved haemoglobin levels in children under 5 years old after 2 years of routine implementation.

## Background

Malaria represents an important public health problem in Africa with the highest morbidity and mortality rates in children under 5 years old [1]. Despite the wide deployment of malaria control interventions, the sub-Saharan Africa region continues to carry a disproportionately high share of the malaria burden, which constitutes a barrier for economic and social development [2, 3]. In Mali, the national prevalence of malaria is 36% in children under 5 years old [4] despite the implementation of malaria control interventions such as intermittent preventive treatment with sulfadoxine-pyrimethamine in pregnancy (IPTp) and long-lasting insecticide-treated bed nets (LLINs) [5].

The World Health Organization (WHO) recently recommended seasonal malaria chemoprevention (SMC) in children under 5 years old, in additional to key interventions for malaria control in sub-Saharan Africa region. It consists to monthly administration of curative dose of sulfadoxine-pyrimethamine (SP) + amodiaquine (AQ) during 4 months in areas with highly transmission season [6]. In Mali, SMC is conducted from July to October according the National Malaria Control Programme (NMCP) recommendation. Beyond its actual efficacy on uncomplicated and severe malaria cases in the target population, this strategy may also impact the whole malaria epidemiology in areas where it is implemented [7, 8]. However, the efficacy of this strategy has been reported in areas where malaria transmission lasts longer only during the SMC period (from July to September) [9], but not after stopped the drug administration in this area where malaria transmission is intense and lasts for 6 consecutive months annually [10].

There is little information on the effect of SMC strategy on malaria epidemiology in areas with long seasonal transmission. Information on malaria trends in these areas is important for the NMCP planning regardless of the variability of malaria endemicity.

In this study, the effect of SMC on malaria was assessed in children during an SMC intervention and 2 months after drug administration was stopped in the village of Dangassa, an area with intense and long seasonal malaria transmission.

## Methods

### Study site

**Fig. 1:**
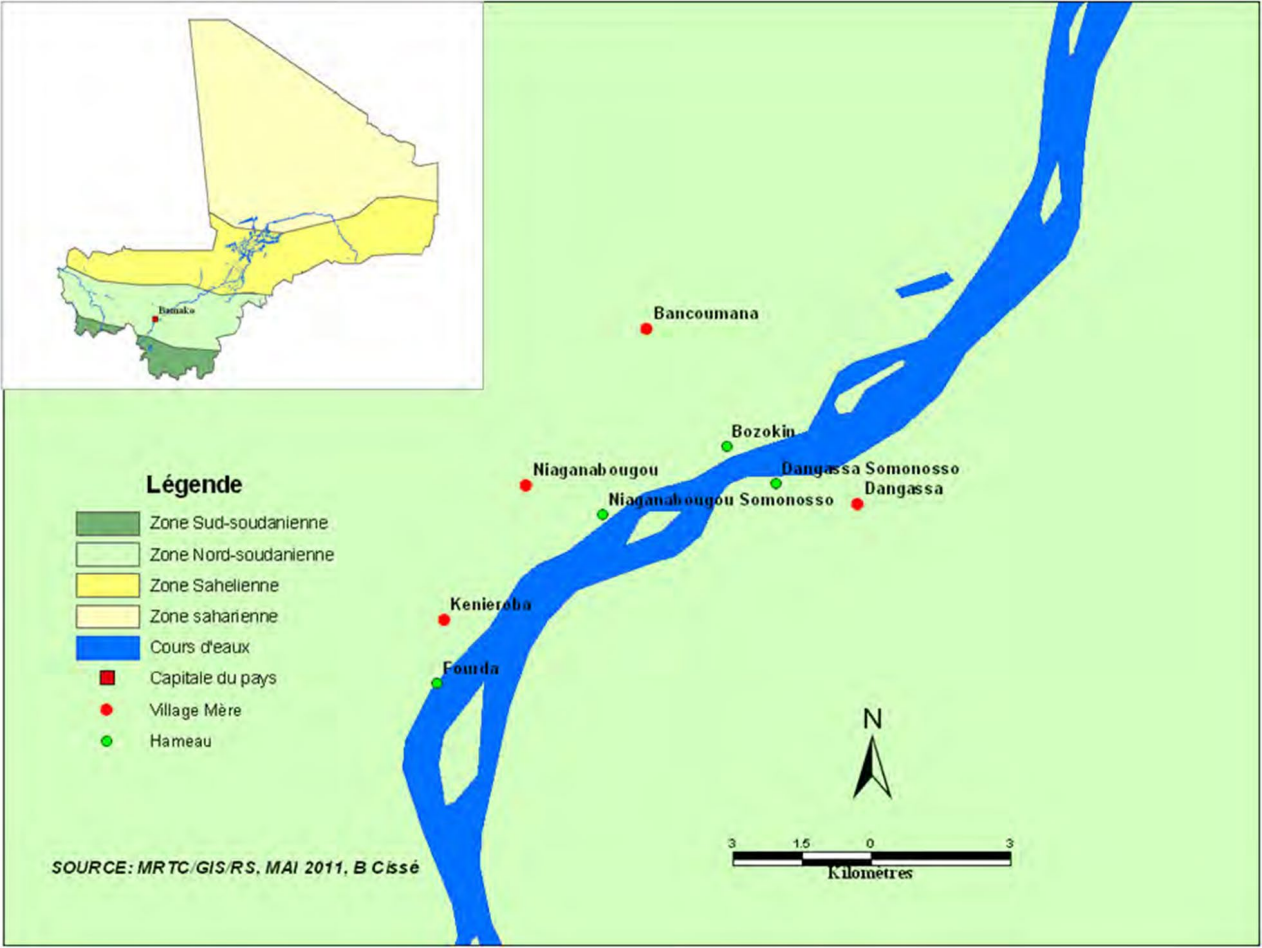
Map of Dangassa village

This study is part of the International Centre for Excellence in Malaria Research (ICEMR) project in West Africa. The main goal of this project was to assess the effect of malaria control interventions on malaria epidemiology. The village of Dangassa is one of the ICEMR project sites in Mali since 2012. Dangassa is located approximately at 80 km southwest of Bamako. The main village is located 4 km from the Niger River (Fig. 1, map of Dangassa village). Malaria transmission is seasonal and occurs during 5 to 6 months per year. The total population of Dangassa is about 6800 inhabitants; Malinke represents the main ethnic group [census data from geographic information system/malaria research & training centre (GIS/MRTC) in 2015]. The study team was based at the community health centre in Dangassa for malaria cases detection.

During the rainy season, mosquito breeding in Dangassa occurs primarily in microhabitats such as the footprints of cattle and natural and man-made pools. During the dry season, as the river is receding, natural pools from the river as well as gold mining pools are formed which serve as breeding sites for mosquitoes, thus extending the transmission season [11].

### Study design and population

Since the start of the ICEMR project in 2012, a dynamic study cohort has been selected from Dangassa population in order to assess the effect of control interventions on malaria epidemiology. This cohort included at least 1400 volunteers randomly selected from the census database with the assumption of an average number of 6 persons per household according demographic and health survey of Mali; about 240 households were selected for the cohort of 1400 children and adults. Children under 5 years of age represented 32.6% of the cohort population. An identification card was designed for all study participants with a unique ID number and a photo ID. Parents of the children were asked to present this identification card during each survey and health centre visit in order to facilitate their identification during the follow-up by the study team. In 2012, 450 children were enrolled in the main cohort and followed yearly for malaria case detection. A new enrolment of children at 3 months of age was done each year before the transmission season for census updated.

Follow-up of participants began in 2013 by the study team based at the Dangassa health centre for passive case detection of malaria. Each participant received at the health centre with malaria symptoms (e.g., temperature > 37.5 °C), a blood sample was taken in order to determine parasitaemia and haemoglobin level. In addition, demographic information (age, gender, ethnic group), and bed net ownership and use (during the consultation at health centre) were collected also. The malaria episode was defined as fever and the presence of *Plasmodium falciparum* in the blood smear using microscopy. Anaemia was defined as a haemoglobin level < 11 g/dl using the Hemocue^®^ 301 device.

### Implementation of routine seasonal malaria chemoprevention

The aim of this study was to determine SMC effect on malaria indicators in children under 5 years old living in Dangassa. During the ICEMR project, a routine SMC was implemented in children under 5 years old during 2015 and 2016 transmission seasons in collaboration with the NMCP. A monthly curative dose of SP + AQ was given to each child in 2015 and in 2016 (SMC) during malaria transmission season (August to October). In this study, 2013 and 2014 were considered as control (2 years prior to SMC implementation = no SMC). In 2015 and 2016, all children under 5 years in the cohort study received three doses of SP + AQ from August to October at the community health centre. The sample size of this study is based on feasibility considerations for both epidemiological and entomological studies assuming a confidence level of 95%. The data from September to December during 2013–2016 malaria transmission seasons were collected on case detection for this study. Before SMC implementation in Dangassa in 2015, the NMCP organized a mosquito net distribution campaign at the start of 2014.

### Data collection and analysis

Data were collected on case report forms (CRF), entered on Microsoft Excel 2017 and analysed using Stata 14. During this study, data were collected on malaria incidence (person/month), fever prevalence, gametocyte prevalence, and haemoglobin level. Cox regression model was used to compare the risk of malaria first episode from September to December during the four malaria transmission seasons with a schedule at 5%. Data from 2014, 2015 and 2016 were compared to those of 2013 (baseline).

## Results

**Fig. 2:**
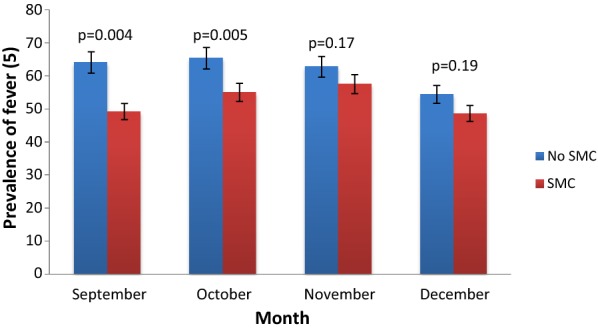
Monthly prevalence of all fever in children under 5 years old in Dangassa from September to December 2013–2016

Fever prevalence decreased in September (49.3%, p = 0.004) and October (55.1%; p = 0.005) during SMC implementation (2015–2016) compared to September and October before SMC. However, it remains similar in November (p = 0.17) and December (p = 0.19), 2 months after stopping drug administration (Fig. 2).

**Fig. 3:**
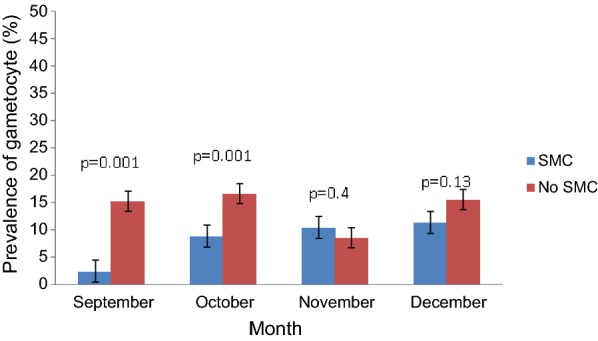
Monthly prevalence of gametocyte in children under 5 years in Dangassa from September to December 2013–2016

A decrease of gametocyte prevalence was observed in September (2.4%; p = 0.001) and October (8.8%; p = 0.001) during SMC implementation compared to September and October 2013–2014 (no SMC) (15.2% and 16.6%, respectively). In November and December, the gametocyte prevalence remains similar SMC vs no SMC groups (Fig. 3).

A significant increase of haemoglobin level was observed in the second year (2016) of SMC implementation in September (p = 0.01), October (p = 0.001) and November (p = 0.001) (Table 1). But it’s was observed only in October and November 2015.

**Table 1:** Hemoglobin level in children less than 5 years in Dangassa from September to December 2013–2016

Years	Hemoglobin level in g/dl			
	September	October	November	December
2013	10.8 ± 0.1	9.8 ± 0.2	10.4 ± 0.2	10.5 ± 0.2
2014	10.2 ± 0.1	10.4 ± 0.1	10.4 ± 0.1	10.7 ± 0.2
2015	10.6 ± 0.1	10.0 ± 0.1	10.8 ± 0.1	10.6 ± 0.1
2016	10.9 ± 0.1	10.7 ± 0.1	10.9 ± 0.1	10.7 ± 0.1

**Fig. 4:**
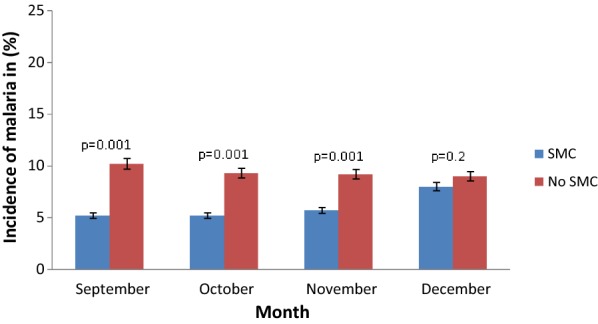
Monthly incidence rate of malaria in children under 5 years from September to December in Dangassa 2013–2016

Malaria incidence decreased from September (5.2 vs 10.2%; p = 0.001), October (5.2 vs 9.3%; p = 0.001) and November (5.7 vs 9.2%; p = 0.001) compared to the same period before SMC implementation. However, malaria clinical incidence remains similar in December (8 vs 9%; p = 0.2), 2 months after stopping drug administration (Fig. 4).

Cox regression model showed after adjusted for insecticide-treated nets (ITN) use, a significant reduction of malaria clinical risk in September (65%, p = 0.001), October (73%, p = 0.001) and November (62%, p = 0.001) in 2015. During the second year of SMC implementation, 75% (p = 0.001) of reduction in malaria clinical risk were observed in September, 77% (p = 0.001) in October and 63% (p = 0.001) in November. However, the risk was similar in December, 2 months after stopping drug administration (p = 0.4, p = 0.5 and p = 0.1) (Table 2).

**Table 2:** Risk of malaria first episode in children under 5 years from September to December 2013–2016 in Dangassa

Variables	September			October			November			December		
	HR	IC 95%	p	HR	IC 95%	p	HR	IC 95%	p	HR	IC 95%	p
Years												
2013	1	(Base)		1	(Base)		1	(Base)		1	(Base)	
2014	0.79	[0.51–1.21]	0.2	0.60	[0.39–0.92]	0.02	0.73	[0.50–10.5]	0.09	0.83	[0.49–1.40]	0.4
2015	0.35	[0.22–0.53]	0.001	0.27	[0.18–0.40]	0.001	0.38	[0.25–0.57]	0.001	0.86	[0.49–1.49]	0.5
2016	0.25	[0.16–0.37]	0.001	0.23	[0.15–0.35]	0.001	0.37	[0.25–0.55]	0.001	0.70	[0.43–1.14]	0.1
ITN USE												
No	1	(Base)		1	(Base)		1	(Base)		1	(Base)	
Yes	1.18	[0.86–1.62]	0.2	1.22	[0.93–1.6]	0.1	1.07	[0.83–1.37]	0.5	1.45	[1.05–1.99]	0.02

## Discussion

The WHO recommends SMC in sub-Saharan Africa Region where malaria season transmission is short and high. In Mali, SMC has been implemented in some health districts since 2012 but a pilot study was conducted in Dangassa village in 2015. The goal of this study was to assess the effects of SMC on malaria trends in children living in Dangassa area were malaria transmission season length is extended by the presence of the Niger River. The data were collected in children enrolled in a main study cohort during malaria case detection from September to December 2013–2016.

Data showed a significant decrease of fever prevalence in September and October during SMC period but remains similar in November and December between SMC vs no SMC (Fig. 2). After two consecutive years of SMC implementation (2015 and 2016), a decrease in gametocyte carriage was observed in September and October in SMC group (Fig. 3). An improvement of haemoglobin level (Table 1) was observed over the intervention period (September to November). The above observations could be attributed to SMC as it has been shown in many other studies about the protective effect of SMC on malaria in children in Africa [12–14]. The regular monthly administration of SP + AQ allows maintenance of drug concentration in blood and prevents parasite growth and avoids red blood cell destruction, which in turn prevents haemoglobin level decrease by malaria parasites [14, 15].

A decrease in malaria incidence was also observed over the drug administration period in both 2015 and 2016. However, a moderate increase in malaria incidence was observed in December, 2 months after the SMC campaign in October in both years 2015 and 2016 (Fig. 4). IPT during the malaria transmission season improved malaria epidemiological trends in children [16]. A substantial reduction of malaria indicators in children was already reported in Mali [8, 12] and elsewhere in Africa [14, 17, 18], which was attributed to SMC implementation in addition to other control interventions, such as IPT and LLINs. The beneficial effect of SMC on malaria clinical incidence has been reported by many other studies [15, 19, 20]. The increase of malaria clinical incidence in December, 2 months after the SMC campaign, could be explained by local epidemiological conditions (proximity to the Niger River and rainfall with abundance of larval breeding) that has an effect on malaria transmission after the rains stop, and also the adherence of population to SMC. Stopping SMC provision while transmission continues could expose children at higher risk because the administrations of drugs to prevent malaria can slowdown the building of their immunity against malaria parasites [10].

The above statements were supported by the results of Cox regression model, which showed a significant reduction of malaria incidence from September to November in both 2015 and 2016 compared to 2013 after adjustment to bed net use. The risk of malaria was similar in 2014, the second year of study cohort without SMC. However, the risk of malaria clinical incidence remained similar in December, 2 months after drug administration was stopped (Table 2). These results suggest the protective effect of SP + AQ on malaria parasite growing [21] but showed that SMC could not have a beneficial effect on malaria trends after stopping drug administration.

The limitation of this study is the not availability of information on other ongoing interventions that could have an effect on malaria trends (e.g., use of ITNs and proper management of malaria cases). Therefore, these results cannot be attributed solely to SMC because it is an additional intervention to others, such as IPTp with SP, the use of ITN (distribution campaign at the start of 2014) and proper management of malaria cases in this area. It would be advisable to develop an appropriate approach for routine implementation of SMC in areas where seasonal transmission of malaria is long and prolonged by the existence of other conditions favouring transmission, as is the case with the Niger River and Dangassa. This is necessary for countries such as Mali where the Niger River and its tributaries cover long distances, in order to prepare targeted interventions necessary for malaria elimination. In addition, a better understanding of the adverse effects associated with amodiaquine metabolism, drug concentration and drug resistance on the systematic implementation of SMC in Mali is essential for its success in malaria-endemic countries.

## Conclusion

This study has shown that SMC implementation in conjunction with other control intervention have significantly reduced malaria indicators in children during SP/AQ drug administration in Dangassa village.

## Data Availability

For any information for the data presented here, please contact the corresponding author.

## Abbreviations

AQ: Amodiaquine
CRF: Case report form
ICEMR: International Center for Excellence in Malaria Research
ITN: Insecticide-treated nets
LLIN: Long-lasting insecticide-treated net
NMCP: National Malaria Control Programme
SMC: Seasonal malaria chemoprevention
SP: Sulfadoxine-pyrimethamine
WHO: World Health Organization
